# Acoustic Comfort Prediction: Integrating Sound Event Detection and Noise Levels from a Wireless Acoustic Sensor Network

**DOI:** 10.3390/s24134400

**Published:** 2024-07-07

**Authors:** Daniel Bonet-Solà, Ester Vidaña-Vila, Rosa Ma Alsina-Pagès

**Affiliations:** HER—Human-Environment Research, La Salle Campus Barcelona—Universitat Ramon Llull, Sant Joan de la Salle, 42, 08022 Barcelona, Spain; daniel.bonet@students.salle.url.edu (D.B.-S.);

**Keywords:** citizen science, acoustic event detection, noise, annoyance evaluation, acoustic comfort, soundscape, WASN

## Abstract

There is an increasing interest in accurately evaluating urban soundscapes to reflect citizens’ subjective perceptions of acoustic comfort. Various indices have been proposed in the literature to achieve this purpose. However, many of these methods necessitate specialized equipment or extensive data collection. This study introduces an enhanced predictor for dwelling acoustic comfort, utilizing cost-effective data consisting of a 30-s audio clip and location information. The proposed predictor incorporates two rating systems: a binary evaluation and an acoustic comfort index called ACI. The training and evaluation data are obtained from the “Sons al Balcó” citizen science project. To characterize the sound events, gammatone cepstral coefficients are used for automatic sound event detection with a convolutional neural network. To enhance the predictor’s performance, this study proposes incorporating objective noise levels from public IoT-based wireless acoustic sensor networks, particularly in densely populated areas like Barcelona. The results indicate that adding noise levels from a public network successfully enhances the accuracy of the acoustic comfort prediction for both rating systems, reaching up to 85% accuracy.

## 1. Introduction

There is a growing interest in the literature to find the best indicators that could be used to accurately evaluate a soundscape to better represent the subjective perception of citizens [1]. The interest in finding the best indicators to evaluate soundscapes stems from the increasing recognition of the importance of the auditory environment in people’s daily lives.

Assessing soundscapes poses unique challenges: for instance, sound perception is highly subjective and can vary greatly among individuals based on their experiences, cultural backgrounds, and personal preferences. Therefore, to accurately evaluate a soundscape, it is important to consider not only physical measurements such as noise levels but also subjective factors that contribute to people’s perception of the acoustic environment.

By identifying and utilizing the best indicators, researchers and policymakers can develop more effective strategies for managing and improving urban soundscapes.

In this work, the authors propose the design of an index capable of predicting the level of acoustic satisfaction of an urban soundscape using a cost-effective approach, consisting of very limited input data as indicators, (i.e., a short audio clip of approximately 30 s and the exact location of the assessed spot, collected in the framework of the research project *Sons al Balcó* [2]), that can be easily obtained with a non-calibrated device such as a smartphone, which is available and easy to use for almost everyone. This simplicity in the process of data collecting makes this approach accessible to a wide range of potential users, from a city council technician aiming to map the acoustic comfort in a neighborhood to a regular citizen searching for a new property who is worried about its acoustic environment.

The specific contribution of this paper is to design a novel and cost-effective approach for predicting the acoustic comfort in urban locations adding objective noise level data obtained from a WASN to the type of sound sources obtained through an automatic sound event detection (ASED) algorithm.

The rest of the paper is organized as follows: Section 2 discusses the related work from other authors and explains the previous work carried out by the authors of the paper. In Section 3, more detailed information on the data used is provided, both for the WASN-based noise indices and for the *Sons al Balcó* contributions. Furthermore, the proposed experimental setup is presented. Next, Section 4 offers the results for the binary assessment and the acoustic comfort index. Section 5 is dedicated to a discussion about the exposed results. Finally, Section 6 summarizes the conclusions for this research study.

## 2. Related Work

In this Section, works from other authors are first analyzed in Section 2.1. Later, the previous work from the authors related to the study presented in this paper is explained in Section 2.2, aiming to contextualize the study.

### 2.1. Several Studies in Literature

A wide variety of approaches have been published by other authors in similar contexts but using different types of data, starting from the use of noise indices obtained from measures performed on-site [3,4,5], using psycho-acoustic parameters [6], taking into account the type of sound events spotted [1,7,8], adding non-acoustic or even non-sensory variables [9,10] or performing a collective assessment in a soundwalk [11] or in laboratory-based experiments [12,13]. Most of these works have been analyzed in [14].

Furthermore, Kang et al. proposed the design of a single index or a set of SoundScape Indices (SSID) [15,16,17] to accurately evaluate the perceived quality of a soundscape. The authors state that an index computed from a wide variety of data, including not only auditory factors such as noise indices, sound source types or psycho-acoustic metrics (loudness, sharpness, etc.) but also non-auditory factors (visual and environmental elements, psychological and other individual features of the people experiencing the soundscapes and other contextual information) would surely give the more accurate assessment possible. However, the amount of information needed for crafting those indices is not always easily available, as it often requires the use of calibrated and expensive equipment operated by experts or it needs an in-depth knowledge of the people who are exposed to the evaluated soundscape.

### 2.2. Previous Work

In previous works, the authors from this paper proposed two different rating systems for predicting the acoustic satisfaction of citizens with the soundscapes around their dwellings [18]. First, a binary evaluation to determine if the soundscape is considered “Positive” or “Negative” was suggested. This first rating system was to be considered a preliminary approach to be complemented with the other rating system, i.e., an acoustic comfort index (ACI) rated on a 5-point scale. The training of the system used data from the citizen science project named *Sons al Balcó* [2], which are the same data used for the present contribution. The proposed ACI tried to predict this perception of the quality of the soundscape using a similar 5-point rating scale (which can be offered as a continuous figure or rounded to mimic the original Likert rating [19]). In that work, the inputs of the system were the acoustic events detected by a convolutional neural network (CNN) that used gammatone cepstral coefficients (GTCC) as inputs.

Even though it achieved accuracy values over 80%, it had a ceiling in the performance due to the limited information that a single short video can offer. The lack of representativeness of the sounds detected was one of several hindrances that affected the accuracy of the prediction [14]. In this present study, a proposal is made to improve the global performance of the estimator using other available information related to the video and the location of the recording.

## 3. Materials and Methods

For this work, different data have been used, both objective and subjective in nature: (1) noise levels measured using a WASN, (2) 30-s videos collected from a citizen science project, and (3) the subjective assessment of soundscapes from urban dwellings obtained from questionnaires related to the aforementioned videos.

Section 3.1 briefly describes the Barcelona noise monitoring network. It also details the calculation of the distance between the evaluated soundscapes and their nearest sensor and why this work only focuses on Barcelona data for the subsequent experimental part. Section 3.2 focuses on the data collected during the 2021 campaign from the emphSons al Balcó project and in the subset of videos and surveys from Barcelona that are used in this present study. Finally, subSection 3.3 outlines the experimental setup.

### 3.1. Sound Sensor Networks

A significant part of the 2021 *Sons al Balcó* contributions were collected in Barcelona (80), which has a sound sensor network deployed. The Barcelona noise monitoring network [20] was initially deployed in 2017 with 60 sound sensors. However, it has been modified through the years. In fact, some of the sensors that were initially installed have already been removed, and many more have been added. During the first semester of 2021, a total of 117 CESVA TA120 Class 1 sound sensors were operating in Barcelona. Most of them were located in spots with clearly defined predominant noise sources, e.g., road traffic noise or leisure activities. More information regarding the exact specifications and placement of the sound sensors is explained in [21].

During the *Sons al Balcó* project, the sensors were operative seven days a week, 24 h a day, even if some of them were affected by technical issues that impacted on the availability of the data. They provided the measured A-weighed sound pressure level (SPL) at 1 min time resolution in the spot.

As the public sensors are placed in specific locations that do not coincide with the places where the *Sons al Balcó* recordings took place, it is important to calculate the distance between the recording points and the sensors. There are different approaches to calculate the distance between two points on the Earth’s surface. For this work, the Haversine formula (Equation (1)) is used, which offers a simple way to obtain a very good approximation in short distances given the coordinates of both locations.

(1) $$d=2R\arcsin\left(\sqrt{\sin^{2}\left(\frac{\phi_{2}-\phi_{1}}{2}\right)+\cos\left(\phi_{1}\right)\cos\left(\phi_{2}\right)\sin^{2}\left(\frac{\lambda_{2}-\lambda_{1}}{2}\right)}\right)$$

In the equation, ϕ is the latitude and λ the longitude (both in radians), and *R* is the Earth’s radius in Barcelona (6368.833 km).

**Figure 1:**
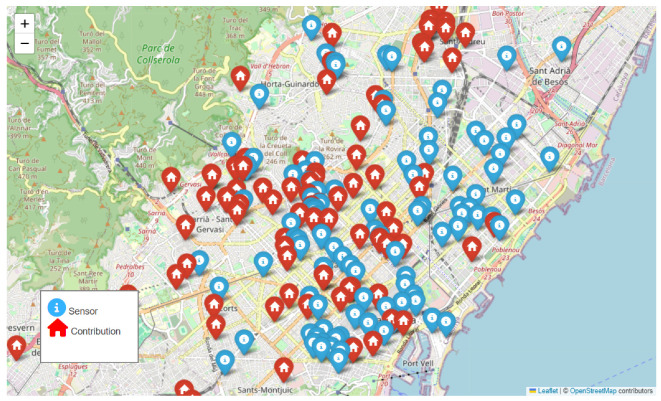
Locations of the Barcelona sound sensor network and the videos collected during the 2021 *Sons al Balcó* campaign.

Figure 1 shows the locations of the sound sensors in Barcelona (black dots) and the locations from which the videos of the 2021 *Sons al Balcó* campaign were recorded (brown dots).

Mean $L_{Aeq}$ from the public sensor network is used for the exact same period of the *Sons al Balcó* 2021 campaign (which collected videos from 20 April to 1 July 2021), with the purpose of having an accurate depiction of the objective noise levels present when the surveys were answered. To test the stability of the system in time and to improve the robustness of the prediction, historical $L_{Aeq}$ has also been acquired. Specifically, measurements for the first semester of 2019 are chosen (historical data from before the COVID-19 pandemic). As $L_{Aeq}$ from 2019 and $L_{Aeq}$ from 2021 are highly correlated (0.95), no significant differences are expected.

**Figure 2:**
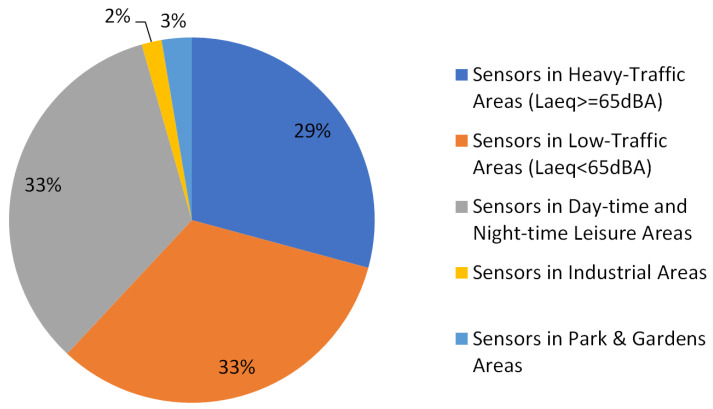
Distribution of the Barcelona sound sensors deployed in 2021 by the predominant noise sources in their locations.

The type of area where the sensors are placed may influence the predictor performance. The percentage of sensors dedicated to each kind of noise source is stated in Figure 2.

Most sensors are located in different areas where there is usually a predominant noise source: road traffic, night-time leisure and industry, as they are the main causes of noise exposure in Barcelona, along with rail [22]. There are also some sensors located in quiet areas with less noise exposure, such as residential areas or parks and gardens.

The heavy-traffic areas are streets where the main noise exposure is related to road traffic. The sensors are usually located in big busy avenues, and the $L_{Aeq}$ measured is high, with mean values over 65 dB. Usually, the soundscapes closer to these streets (and their sensors) are more deteriorated and they improve with the distance.

The low-traffic areas have mean $L_{Aeq}$ values under 65 dB. They usually have a lower road traffic exposure and better quality soundscapes. It is more difficult to predict whether the soundscapes will remain good or will worsen with the distance to the sensor as there may be areas with high-traffic surrounding the residential areas. The same happens with the park and garden areas. However, park and garden sensors are more unreliable as these areas are sometimes used as spots for leisure activities that can also be annoying, especially at night-time.

The sensors located in day-time and night-time leisure areas are usually situated in city squares in the middle of residential areas or in the Old City Center nearby touristic spots. They usually register lower $L_{Aeq}$ levels than those located in heavy-traffic areas. However, the noise related to the nightlife can be especially annoying even if the mean $L_{Aeq}$ is lower. Normally, the soundscape quality is very poor near the sensor but rapidly improves with the distance.

Industrial areas are usually located in the outskirts of the city, far from most residential areas. That is the main reason why only a small percentage of citizens are exposed to this kind of noise.

The proposed experiments use $L_{Aeq}$ levels, the type of area where the sensors are placed and the distance between the sensor and the location of the recording to try to predict the subjective assessment of a dwelling’s soundscape.

### 3.2. *Sons al Balcó* Data

*Sons al Balcó* is a citizen science project that launched several campaigns starting in 2020 during the lockdown caused by the COVID-19 pandemic to encourage the general population across Catalonia to participate in a multi-disciplinary study of the soundscape quality in their dwellings. They were asked to send a short 30 s video (including audio) recorded from their balconies and to answer a survey about their perception of the soundscape’s quality and sound events. More specifically, the participants were instructed to record a video using their own smartphones. The video had to be representative of the typical soundscape around their dwelling. They had to shoot from a balcony or window, keeping silence and being careful not to cover the microphone during the recording. The meta-data collected included the exact geographic location where the videos were recorded.

During the campaign performed in 2020, 365 valid videos were received. However, as they were recorded during the lockdown, with severe mobility restrictions and activity regulations, the subjective assessment was unanimously positive and unfitting for the purpose of this research. On the contrary, the 237 videos collected during the back-to-normal post-pandemic scenario in 2021 (from 19 April 2021 to 16 July 2021) were differently assessed, including a diversity of negative, neutral and positive scenarios, making them suitable for the present work. More information on the data collected during the 2021 *Sons al Balcó* campaign can be found in [23].

**Figure 3:**
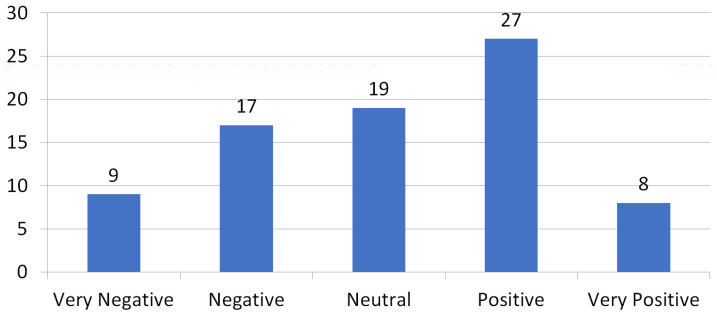
Subjective Assessment of the Soundscapes in Barcelona (Likert scale).

The subjective assessment of the soundscapes across Catalonia was still significantly biased towards positive scenarios [14]. The subset of 80 videos that was recorded in Barcelona, on the other hand, has a more balanced distribution in the global assessment of the soundscapes, as shown in Figure 3.

### 3.3. Experimental Setup

This subsection explains the different experimental set-ups, both for the binary and the 5-points assessments.

#### 3.3.1. Binary Assessment

**Figure 4:**
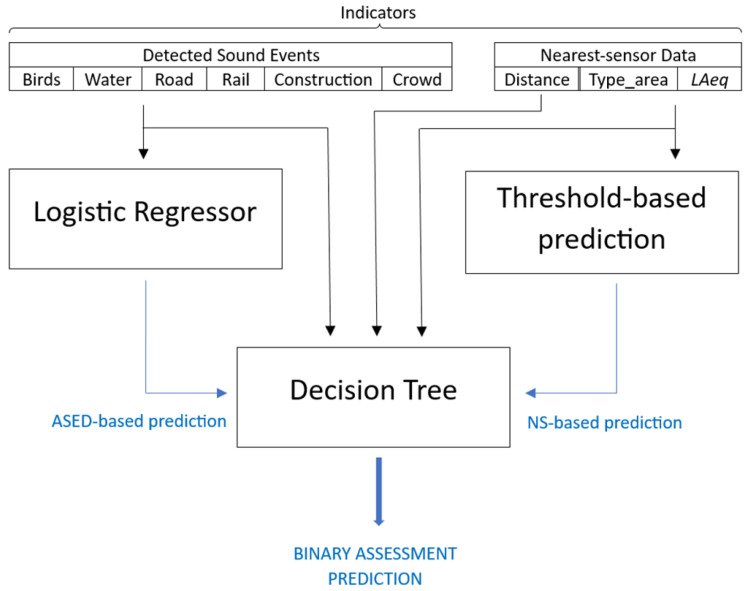
Enhanced Dwelling’s Soundscape Quality Estimator (Binary).

In the first stage, a straightforward predictor only based on the data obtained from the nearest sensor to the location where each video was recorded is designed (corresponding to the threshold-based prediction block in Figure 4). This predictor offers a binary assessment classifying each dwelling as having a “Positive” soundscape or a “Negative” soundscape. This specific prediction is based on the $L_{Aeq}$ obtained from the nearest sensor available (taking into account the type of area, i.e., the predominant noise source stated in Figure 2, where the sensor is located, as recommended in [24]).

Next, the ASED-based prediction that was developed in a previous work by the authors [14] is added (corresponding to the logistic regressor in Figure 4). Finally, a decision tree (DT) is implemented. This DT uses as inputs the type of sounds detected by the ASED algorithm, the distance between the sensor and the studied soundscape and the type of area where the sensor is circumscribed to choose one of both predictions, i.e., the ASED-based prediction or the nearest-sensor-based (NS-based) prediction, according to the expected reliability of each one (decision tree block in Figure 4).

To validate the DT design, the dataset of available videos was divided into four folds and, subsequently, a 4-fold cross-validation scheme was implemented. The metrics used to assess the results of this binary estimator are Accuracy and F1-Score.

#### 3.3.2. Acoustic Comfort Index

In the second stage, another predictor also based on data obtained from the nearest sensor to the assessed soundscape’s location is also implemented. However, this time it outputs a 5-point scale figure. This new predictor is designed as a linear regressor, which uses the $L_{Aeq}$ provided by the sensor, the type of area where the sensor is circumscribed and the distance between the sensor and the location of the dwelling as inputs.

**Figure 5:**
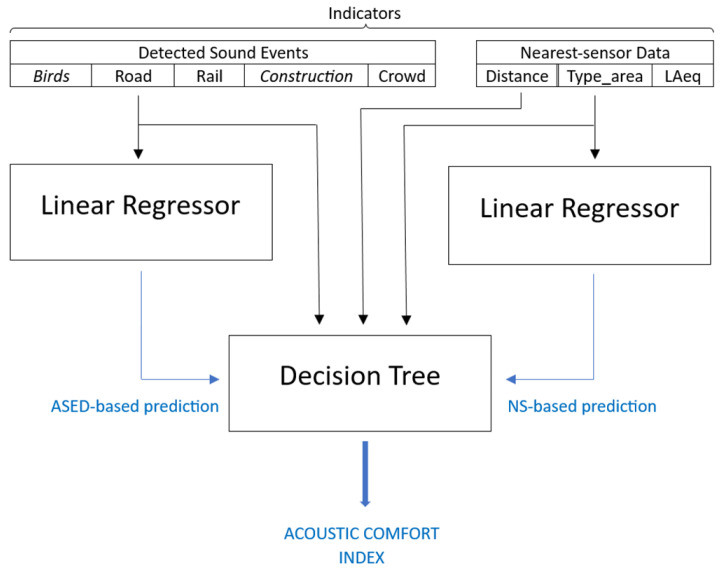
Enhanced Dwelling’s Acoustic Comfort Index Estimator.

Subsequently, this NS-based prediction is combined with the 5-point scale assessment obtained from the ASED-based predictor [14]. They are combined using two approaches: (1) Using a decision tree (Figure 5) also validated through a 4-fold cross-validation scheme and (2) calculating the mean value of both predictions, i.e., the ASED-based prediction and the NS-based prediction.

In this case, the decision tree uses some of the sound categories detected by the ASED algorithm, i.e., road, rail and crowd, along with the indicators used by the NS-based estimator as input features. A separate study will also be conducted with rounded 1–5 point predictions (1 for “Very Negative”, 2 for “Negative”, 3 for “Neutral”, 4 for “Positive” and 5 for “Very Positive”) in order to mimic the Likert scale used by participants in the *Sons al Balcó* project to assess the soundscape.

This 5-point-scale assessment will be named the acoustic comfort index (ACI) from now on, as proposed in [14]. In order to evaluate the performance of this ACI, two different error metrics are computed: the mean absolute prediction error (MAPE) (Equation (2)) and the root mean square prediction error (RMSPE) (Equation (3)):

(2) $$MAPE=\frac{\sum_{i=1}^{n}\left|y_{i}-{\hat{y}}_{i}\right|}{n}$$

(3) $$RMSPE=\sqrt{\frac{\sum_{i=1}^{n}{\left(y_{i}-{\hat{y}}_{i}\right)}^{2}}{n}}$$

To assess the specific case of the rounded ACI, a ±1 interval accuracy is also calculated.

## 4. Results

In Section 4.1, the results for the binary estimator of the perceived quality of a dwelling’s soundscape are presented. Next, Section 4.2 is dedicated to the results for the 5-point scale predictor (acoustic comfort index).

### 4.1. Binary Assessment Results

#### 4.1.1. Binary Nearest-Sensor-Based Estimator

The basic NS-based estimator is grounded in the hypothesis that the subjective rating of an urban soundscape will generally be negatively correlated with the noise levels in the surroundings. For the first experiment, the noise indices used are those provided by Barcelona’s WASN concurrently to the 2021 *Sons al Balcó* campaign (20 April 2021–1 July 2021). However, the system has also been tested using historical data from the first semester of 2019 in order to test the robustness and independence from a specific timeframe of sensor data. There is a strong positive correlation between mean $L_{Aeq}$ and mean daily indices ($L_{d}$, $L_{e}$, $L_{n}$) dynamics in the studied sensors (between 87 and 97%). The prediction obtained by the estimator would be effectively the same regardless of the chosen index. For simplicity, mean $L_{Aeq}$ has been chosen as the default input to the predictor.

As shown in Figure 4, a threshold-based prediction is implemented. As relationships between sensor data and subjective appraisals depend on the predominant noise source [24], a customized $L_{Aeq}$ threshold has been chosen depending on the type of area where the sensor is circumscribed, i.e., heavy-traffic, low-traffic, leisure or park. These thresholds have been optimized to maximize the accuracy of the prediction by performing a grid-search. For sensors located in heavy-traffic areas, the optimal threshold was 65.2 dB. On the contrary, for sensors located in low-traffic areas, the best performing threshold was 62.7 dB. In leisure areas, any threshold between 65 and 66 dB achieved the best accuracies. Finally, the few sensors located in parks or public gardens gave a low reliability in the binary assessment prediction regardless of the selected threshold. Therefore, a threshold of 65.2 dB has been chosen for all types of areas, except for low-traffic zones where 62.7 dB was preferred.

The distance between the studied dwelling and its nearest sensor is a significant factor that must be considered. For some contributions that are too distant from their nearest sensor, it is advisable to discount noise indices as a reliable predictor. That being said, as the available sample of contributions in Barcelona is limited, it is important to maximize the number of viable candidates. Of the total of 80 videos collected in Barcelona, 19 are qualified as neutrals and, therefore, not apt for the binary assessment. Another one is discarded for being too short (less than 8 s) for comparison purposes with the baseline ASED-based estimator [14]. For the remaining 60, most of them are not excessively distant from a sensor (more than two thirds are less than 500 m apart from their nearest sensor). However, dwelling–sensor distances fluctuate between 25 m and 2 km.

**Figure 6:**
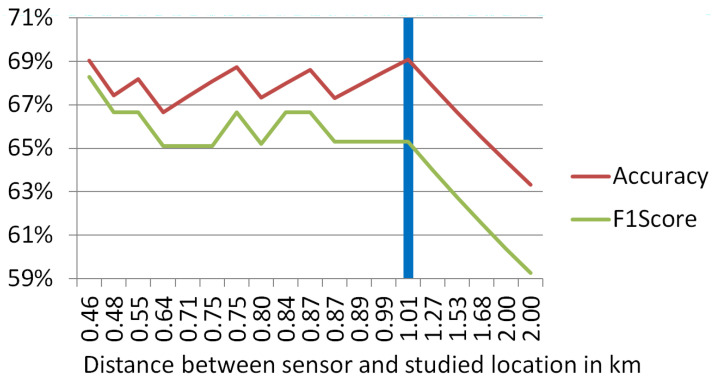
Performance of the nearest sensor-based prediction depending on the maximum distance between sensor and studied location accepted.

In Figure 6, accuracy and F1-Score were computed, including only those instances compliant with a given soundscape-sensor distance. Grounded on the previous work [24], which confirmed the alignment between sensor data and subjective perception of the soundscapes in dwellings up to 1 km distant, a grid-search for accepted distances was performed from just under 500 m to 2 km. For the first km, the accuracy fluctuates between 67% and 69%, and F1-Score also fluctuates between 65% and 68%. These oscillations are mostly caused by the limited size of the dataset. At approximately 1 km, the fluctuations stop, and both metrics start to fall steadily with distance. Accuracy drops to just over 63% and F1-Score descends to just over 59%. These drops are especially significant taking into account that there are very few soundscapes located farther than 1 km from their nearest sensors. Consequently, 1.01 km is the maximum permitted distance for this present study. From the 60 soundscape candidates, 55 are included in the accepted 1.01 km range.

Table 1 compares the accuracy and F1-Score for the prediction of this subset of soundscapes using the NS-based approach with the performance obtained using the ASED-based approach for the same subset of videos.

The ASED-based binary prediction for the subset of Barcelona contributions achieves a very similar accuracy when compared to the whole Catalonia-wide dataset [14], as seen in Table 1. However, F1-Score is greatly improved (from 64.15 to 78.43%). This F1-Score increase is explained by the more balanced subjective assessment of Barcelona’s soundscapes compared to the more positively biased Catalonia scenario. In Barcelona, discarding neutrally assessed soundscapes, 41.07% of the dwellings were rated as “Negative” or “Very Negative”, as opposed to the 58.93% that were rated as “Positive” or “Very Positive” (Figure 3). On the contrary, including all surveys across Catalonia, only 25.77% of the dwellings were rated negatively, and 74.23% were rated positively [23]. In fact, for the Barcelona subset, accuracy and F1-Score are especially close.

The performance achieved with the NS-based approach for the binary prediction is significantly lower than the performance of the ASED-based implementation. The accuracy for the NS-based predictor is more than 10% lower and the F1-Score’s drop is even higher, exceeding 13%. This poorer performance was expected because noise indices alone are limited to explain subjective appraisal of a soundscape [25], especially when noise sources are different from road traffic. In addition, the sensors are not located in the exact same spot as the assessed dwellings. Thus, they are bound to be less reliable than the prediction based on sound events present at the precise location being evaluated.

As it has already been stated, it may be difficult to access concurrent sensor data from a WASN. For that reason, it is interesting to evaluate the performance of the predictor using also historical data. In this case, Table 2 shows the results of both the ASED-based approach and the NS-based approach when 2019 sensor data are being used. The subset of valid candidates is slightly different because in 2019 there were only 70 active sensors, and only 53 of the 60 eligible soundscapes were circumscribed inside the accepted 1.01 km distance from sensors. As observed in Table 2, the results when historical data are being used are comparable to the ones presented in Table 1 with concurrent sensor data, even slightly better. That proves that the predictor is not dependent on the contemporaneity of noise levels if there is not a drastic change in the soundscape composition of the surroundings.

Although it is clear from the Table 1 and Table 2 results that the NS-based prediction is not recommended per se, it can be used conjointly with the ASED-based prediction to increase the final performance without needing specific calibrated measurements performed on-site, as is further explored in Section 4.1.2.

#### 4.1.2. Enhanced Binary Dwelling’s Soundscape Quality Estimator

As a preliminary analysis, it is interesting to observe how many of the soundscapes are equally predicted by both estimators, i.e., ASED-based and NS-based, and what is the achieved performance for this subset. From the 55 videos predicted using both approaches, 34 gave identical results. From these, only four were errors (one false positive and three false negatives), giving an accuracy of 88.24%. This accuracy hints at the potential ceiling of this enhanced binary soundscape quality estimator.

As shown in Figure 4, a decision tree was implemented to decide which one was the more reliable answer among the two approaches. Understandably, this decision tree can only be applied when data from a sensor located within a 1.01 km are available.

Choosing the input features for the decision tree is grounded in two assumptions. The first premise consists of the fact that the ASED-based approach does not offer the same results depending on the type of sound event detected. In fact, a preliminary study conducted in [14] by the authors already determined that birds are homogeneously considered agreeable sound events. Thus, it is reasonable to expect that videos where only birds are detected are correctly predicted as “Positive” with a higher-than-average accuracy. In contrast, presence of other noise sources such as road traffic or leisure noise are not always deemed annoying by citizens. This has a negative impact in the accuracy for this subset of videos. The second premise is that the type of area where the sensor is circumscribed [24], e.g., heavy-traffic or leisure, and the distance between the sensor and the dwelling’s location also have an effect on the prediction’s reliability.

Consequently, with the two previous premises, the input features chosen for the decision tree are as follows: the type of sound event detected, i.e., **birds**, **water**, **road traffic**, **rail**, **construction** and **leisure**; the type of area of the sensor, i.e., **heavy-traffic**, **low-traffic**, **leisure** area and **park**; and the soundscape-nearest sensor distance. A 4-fold cross-validation scheme has been implemented with 75% of the contributions dedicated to train and 25% dedicated to test. The decision tree is set to predict two targets: the reliability of the ASED-based prediction and the reliability of the NS-based prediction. After that, the algorithm chooses for each tested soundscape the prediction with a higher probability of success according to the outputs of the decision tree.

The final results achieved by the enhanced soundscape quality estimator depicted in Figure 4 are an accuracy of 85.44% and a F1-Score of 83.33% (Table 3). Both metrics show approximately a 5% improvement compared to the performance achieved by the original ASED-based predictor (Table 1). If the 2019 historical data are used, comparable results are obtained (as shown in Table 3), proving again the independence of the predictor from the exact timeframe of the measurements.

### 4.2. Acoustic Comfort Index Estimator

Following the same structure used for presenting the binary assessment results, Section 4.2.1 presents the results of the NS-based approach for the ACI estimator. Next, Section 4.2.2 integrates this NS-based estimation with the original ASED-based estimation to obtain an improved final prediction, and Section 4.2.3 offers the results for the rounded ACI estimator.

#### 4.2.1. ACI NS-Based Estimator

According to the design proposed in Figure 5, a linear regressor was implemented with several input features: mean $L_{Aeq}$ obtained from the nearest sensor, type of area where the sensor is located and soundscape-sensor distance. The mean absolute prediction error (MAPE) achieved with this proposal is 1.01 points, worse than the 0.84 points achieved with the ASED-based estimator. These results were tested with the subset of 73 contributions received from Barcelona that were included in the accepted distance range from the sensors. Focusing on another metric, the RMSPE error, the NS-based approach also performs worse (1.18 points) than the ASED-based approach (1.04 points).

The mean error computed for the NS-based implementation depends on the type of area where the sensor is placed, as stated in Table 4. It performs better in quieter areas such as parks, public gardens or residential neighborhoods where the $L_{Aeq}$ measured is usually lower.

#### 4.2.2. Enhanced ACI ASED+NS-Based Estimator

Two different approaches were implemented in order to integrate the ASED-based predictions and the NS-based predictions in the 5-point scale acoustic comfort index estimator. The first approach consisted of averaging both original predictions to give the final ACI figure. For the second approach, a decision tree was used to choose between both estimations, as seen in Figure 5.

The selected input features for the decision tree are very similar to the ones used in the binary estimator and already described in Section 4.1.2, i.e., the type of area, the $L_{Aeq}$ provided by the nearest sensor, the soundscape-sensor distance and some of the detected sound events. In this particular case, **birds** and **construction** have been finally removed as they did not improve the performance of the integrated estimator.

**Figure 7:**
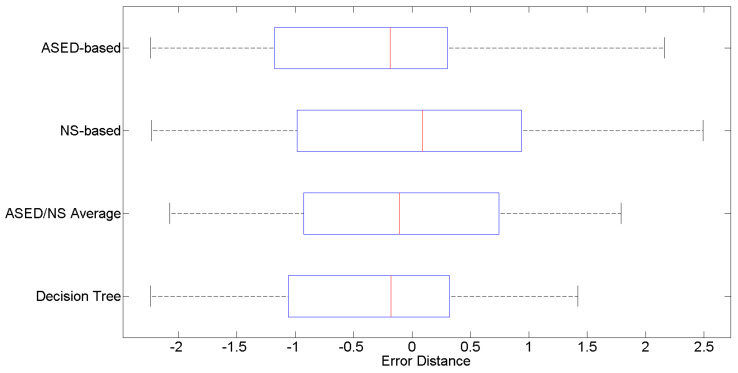
Error distance for the ACI assessment for the ASED, nearest-based and combined approaches.

Figure 7 offers a comparative of the error distance for each individual prediction using the ASED-based estimator, the NS-based estimator and both integrated approaches: averaging and decision tree. A negative error distance indicates that the acoustic comfort predicted is exceedingly positive compared to the real opinion expressed by the dwellers and otherwise.

Even though there is not a substantial difference between the different approaches, the ASED-based prediction is significantly biased in the Barcelona subset of dwellings. It tends to give a better rating to the soundscapes than they deserve. Median is slightly negative and the second quartile is clearly wider than the third one. It has already been stated that the percentage of negatively reported soundscapes in Barcelona is higher than in the rest of Catalonia and the ASED-based predictions are usually poorer for negatively appraised soundscapes [14]. On the other hand, error distances are higher for the NS-based predictor, but the bias between positive and negative acoustic environments is almost nonexistent.

Averaging both predictions partially corrects the bias and offers error distances with only a slight increase compared with the ASED-based scenario. Both the second and third quartiles are within the ±1 error distance range. Moreover, the first and fourth quartiles are approximately within the ±2 error distance range. The RMSPE error for this approach is 0.42% lower than the error made by the ASED-based design (see Table 5). In spite of this, MAPE error is higher (0.89) than in the baseline predictor (0.85). Finally, the combined method based on a decision tree is the approach that achieves better results according to the different error metrics. The RMSPE is almost the same as in the averaged predictor. Furthermore, the MAPE error decreases to 0.835, which is a 1.14% improvement compared to the ASED-based estimator. It should be noted, though, that the decision tree design retains the original bias of the ASED-based predictor because, in general, it favors ASED-based estimations over NS-based estimations (as is logical given the original performance of them).

When 2019 historical data are used for the NS-based prediction, the integrated decision tree approach achieves even better results, as seen in Table 5. MAPE decreases to less than 0.8 (which means an improvement of 6.81%) and the RMSPE falls below 1 (5.06% improvement). On the other hand, the averaged solution only offers a modest drop for the RMSPE with a slight 0.16% improvement.

Table 5 also includes the R-squared values for the baseline ASED-based prediction and for the two combined estimators. In general, the option based on the decision tree is clearly recommended as it improves the results for the three studied metrics (MAPE, RMSPE and R2) not only when using 2021 sensor data but also with the 2019 noise levels.

#### 4.2.3. Rounded ACI Assessment

The rounded ACI estimator is based in the same scheme proposed in Figure 5. The only difference is that, in this case, birds and construction remain being used as input features as opposed as what happened in the not-rounded predictor. In this particular case, they were not detrimental to the estimator’s performance. As for the averaged solution, the average is computed before rounding the final result.

In this case, the averaged proposal gives worse results than the baseline ASED-based approach. On the contrary, the solution based on a decision tree is, again, the preferred implementation as it improves on all metrics for both concurrent and historical data, as seen in Table 6.

Focusing on the percentage of predictions inside the ±1 prediction interval, it is confirmed that adding information from the nearest sensor positively affects the accuracy of the final estimator (from 82.19% to 84.93% using concurrent sensor data and even higher using historical data).

## 5. Discussion

The proposed solution continues to offer a simple and non-expensive method to assess the perceived quality of a dwelling’s soundscape. It still only requires a short video or audio clip of approximately 30 s that can be recorded using a smartphone like in the original predictor [14] proposed by the authors. The only additional information needed is the GPS location of the recording, which is immediately available in any standard smartphone and a database with sensor locations and $L_{Aeq}$ levels that in many cases is publicly accessible and does not require being up-to-date.

The main limitation of the present study is that from the 237 surveys answered during the 2021 campaign of *Sons al Balcó*, only the subset of 80 volunteers living in Barcelona could be used for this study. It is the only city in Catalonia where the deployed WASN is big enough to ensure available sensor data in the proximity of most of the assessed soundscapes. Even though the number of participants included is reduced, they come from all the districts within the city and are located in areas with different predominant noise sources.

Barcelona is a metropolis with high levels of noise pollution. The authors already proved in [14] that the size of the city has an effect on the ASED-based prediction. It seems fair to assume that the results presented in this work could change in less populated cities. However, small cities or towns usually do not have a deployed WASN permanently active, and the estimator proposed in this work is only applicable with an existing network of acoustic sensors. That being said, the original ASED-based prediction would still be an optimal solution in those places where a WASN is not available.

Only 8 of the 55 soundscapes evaluated using the binary rating were incorrectly predicted, as seen in Table 7. Six of these errors already existed with the original ASED-based prediction, but the other two can be ascribed to the decision tree algorithm failing to choose the right option. On the other hand, five errors made by the ASED-based estimator (45.45% of the total) have been fixed with this enhanced proposal. Therefore, adding information from the nearest sensor to the original predictor is clearly justified and recommended, even if it has only been tested in a limited sample of soundscapes.

The confusion matrix in Table 8 confirms that the majority of the predictions for the rounded ACI are either exact or inside a ±1 interval. It also shows that predictions tend more to a “Neutral” rating than a “Very Positive” or “Very Negative” one and are slightly biased towards giving higher ratings than lower. In fact, from the 11 exceptional instances where the error falls outside the ±1 interval, 9 correspond to “Very Negative” or “Negative” soundscapes incorrectly predicted as “Neutral” or “Positive”, respectively. There is not a single prediction with more than two points of error.

**Figure 8:**
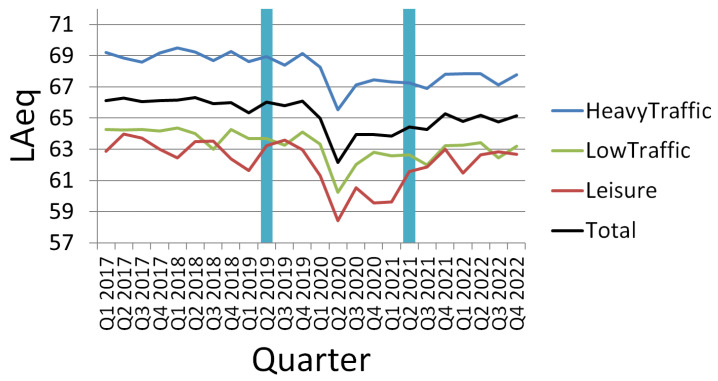
Quarterly evolution of the $L_{Aeq}$ measured in the subset of BCN sound meters network used during this present study that were continuously collecting data from 2017 to 2022.

Even though the performance obtained by the enhanced ASED + NS predictor clearly improves the results of the original ASED-based one when data from 2021 are used, the improvement is even more significant when historical data from 2019 are chosen. That can be better understood observing the quarterly evolution of the mean $L_{Aeq}$ measured in the sensors used for this study (Figure 8).

Data from a total of 42 sensors for the Barcelona noise monitoring network were finally used (those that were chosen for their proximity to the assessed soundscapes). The mean $L_{Aeq}$ measured in those 42 sensors during the first semester of 2019 was 66.04 dBSPL(A). In contrast, during the first semester of 2021, the mean level for the same set of sensors was 64.2 dBSPL(A), almost 2 dB lower. In fact, two sensors that in 2021 were located in low-traffic areas (with mean $L_{Aeq}$ levels inferior to 65 dBSPL(A)) were considered heavy-traffic in 2019.

In Figure 8, the evolution of the quarterly mean $L_{Aeq}$ level for the sensors that were continuously working from 2017 to 2022 is depicted. The graph shows not only the noise levels for the aggregated set of sensors (in black) but also the quarterly mean $L_{Aeq}$ levels for the subsets of sensors located in different kinds of areas, i.e., heavy-traffic, low-traffic and leisure. The few sensors located in parks were not yet operative in 2017 and thus were not included in the Figure. Vertical bars mark the quarters from where levels for the present study were obtained. It can be observed that levels before the lockdown (first quarter of 2020) were stable (even though sensors located in leisure areas experience a seasonal variation with periodic drops during the winter months). In 2020, all noise levels experienced a steep decrease caused by the restrictions imposed during the COVID-19 lockdown [21]. From 2021 on, noise levels have been slowly growing again but, as seen in Figure 8, they have not yet reached the original pre-pandemic scenario. That is especially true for the first semester of 2021, where noise levels are considerably lower than those registered from 2017 to 2019. Therefore, for citizens inhabiting the same dwelling for a long time, pre-lockdown data could better represent their perception on soundscape’s quality than noise levels obtained during the 2021 *Sons al Balcó* campaign.

It is worth mentioning that even though only the data from the nearest sensor to the dwelling were finally used; experiments were also conducted using data from the next *k*-sensors included within the 1.01 km range. However, the increased complexity of this approach was not justified as it did not improve the global performance. Thus, it was finally discarded.

The results produced using the proposed approach surpass those obtained from other published works. The only other previous study that used the same data-set [14] achieved 80% accuracy for the binary estimation and 82% accuracy for the rounded ACI in the Barcelona subset of contributions. In contrast, this novel approach increases the accuracy to 85% in both cases. Other works normally use different indicators to predict perceptual constructs related to noise annoyance and acoustic satisfaction. The metrics used are also heterogeneous and often difficult to compare. For example, a previous work by Kang et al. [7], which also uses ASED as a predictor for several perceptual constructs, concludes that automatically detected sound events account for 57.5% of the variability of the studied constructs.

## 6. Conclusions

The enhanced binary predictor combining both ASED-based and NS-based estimations significantly improves the accuracy and F1-Score of the original baseline proposal [14]. The system is independent from the time-frame where sensor data are collected, provided that there is not an exceptional event such as the lockdown. For that reason, historical sensor data can be used. That is one of the strengths of the proposed estimator, as it does not rely on the concurrent acquisition of noise levels. It achieves accuracies over 85% for prediction subjective assessments using only a short (30 s) audio clip and the GPS coordinates of the recording.

As for the ACI prediction, the minimum error and the better prediction are also achieved when a decision tree is implemented to choose among the original ASED-based estimation and the NS-based approach. The ±1 accuracy interval includes close to 85% of the predictions, also significantly improving the performance for the initial prediction where only sound events detected were taken into account.

The viability of the proposed enhanced dwelling’s acoustic comfort predictor relies on the existence of a network of acoustic sensors deployed in the assessed city. There are a growing number of cities with WASNs deployed. However, they are still usually restricted to big urban settlements.

The number of available surveys for Barcelona is limited and it is advised to test the predictor with further data from future campaigns. In spite of that, the results have been validated using a 4-fold cross-validation scheme. It would also be interesting to test the system with data obtained from different geographic and cultural contexts as it has been acknowledged that contextual non-sensory aspects affect the perceptual appreciation of the soundscapes [26].

As a future work, it could be interesting to also add automatically detected visual elements to see if the type of landscape constituents also has a significant effect on the prediction.

## Figures and Tables

**Table 1:** Performance comparative between the ASED-based approach and the NS-based approach using concurrent sensor data from 2021 (binary assessment).

	Accuracy	F1-Score
ASED-based prediction (Catalonia) [14]	80.41%	64.15%
ASED-based prediction (only videos less than 1.01 km from nearest sensor)	80%	78.43%
NS-based prediction (only videos less than 1.01 km from nearest sensor)	69.1%	65.31%

**Table 2:** Performance comparative between the ASED-based approach and the NS-based approach using 2019 historical data from sensors (binary assessment).

	Accuracy	F1-Score
ASED-based prediction with 2019 data (only soundscapes less than 1.01 km from nearest sensor)	81.13%	80%
NS-based prediction with 2019 data (only soundscapes less than 1.01 km from nearest sensor)	69.81%	66.67%

**Table 3:** Performance of the enhanced dwelling’s soundscape quality estimator using 2021 concurrent data and 2019 historical data from sensors (binary assessment).

	Accuracy	F1-Score
ASED + NS-based prediction with 2021 data (only soundscapes less than 1.01 km from nearest sensor)	85.44%	83.33%
ASED + NS-based prediction with 2019 data (only soundscapes less than 1.01 km from nearest sensor)	85.03%	83.33%

**Table 4:** Mean Error for the ACI assessment depending on the type of area where sensors are located.

Type of Area Where Sensor Is Located	MAPE	RMSPE
Heavy-Traffic Area	1.13	1.29
Low-Traffic Area	0.82	1
Leisure Area	1.06	1.24
Park or Garden Area	0.84	0.93

**Table 5:** Error metrics comparative for the baseline ASED-based approach and both integrated proposals using concurrent and historical data (not-rounded acoustic comfort index). Only contributions with a soundscape-sensor distance inferior to 1.01 km are included.

		2021			2019	
Prediction Approach	MAPE	RMSPE	R2	MAPE	RMSPE	R2
ASED-based	0.845	1.037	0.308	0.854	1.047	0.311
ASED + NS (Averaged)	0.893	**1.033**	0.293	0.910	1.045	0.287
ASED + NS (Decision Tree)	**0.835**	**1.033**	**0.333**	**0.796**	**0.994**	**0.377**

**Table 6:** Error metrics comparative for the baseline ASED-based approach and both integrated proposals using concurrent and historical data (rounded acoustic comfort index). Only contributions with a soundscape-sensor distance inferior to 1.01 km are included.

		2021			2019	
Prediction Approach	MAPE	RMSPE	±1 Interv. Accuracy	MAPE	RMSPE	±1 Interv. Accuracy
ASED-based	**0.822**	1.085	82.19%	0.808	1.079	82.19%
ASED + NS (Averaged)	0.931	1.123	83.56%	0.944	1.132	80.82%
ASED + NS (Dec. Tree)	**0.822**	**1.06**	**84.93%**	**0.781**	**1.027**	**86.3%**

**Table 7:** Confusion Matrix for the Binary Assessment.

	Actual Negative Values (0)	Actual Positive Values (1)
Predicted Negative Values (0)	27	3
Predicted Positive Values (1)	5	20

**Table 8:** Confusion Matrix for the ACI Assessment.

Predicted			Actual Values		
Values	Very Negative	Negative	Neutral	Positive	Very Positive
Very negative	0	0	0	0	0
Negative	2	2	0	1	0
Neutral	7	10	9	11	1
Positive	0	2	9	13	6
Very positive	0	0	0	0	0

## Data Availability

No new data were created or analyzed in this study. Data sharing is not applicable to this article.

## Abbreviations

The following abbreviations are used in this manuscript:

ACI	Acoustic comfort index
ASED	Automatic sound event detection
CNN	Convolutional neural network
DT	Decision tree
GTCC	Gammatone cepstrum coefficients
IoT	Internet of Things
$L_{Aeq}$	A-weighted equivalent sound level
$L_{d}$	Day noise level. A-weighted Leq, over the 14-h day period (7 to 21)
$L_{e}$	Evening noise level. A-weighted Leq, over the 2-h evening period (21 to 23)
$L_{n}$	Night noise level. A-weighted Leq, over the 8-h night period (23 to 7)
MAPE	Mean absolute prediction error
NS	nearest sensor
RMSPE	Root mean square prediction error
SPL	Sound pressure level
SSID	Soundscape indices
WASN	Wireless acoustic sensor network
